# Involvement of IGF-1R-PI3K-AKT-mTOR pathway in increased number of GnRH3 neurons during androgen-induced sex reversal of the brain in female tilapia

**DOI:** 10.1038/s41598-022-06384-4

**Published:** 2022-02-14

**Authors:** Akari Oda, Sakura Inoue, Ryo Kaneko, Yasuto Narita, Suzuka Shiono, Toyoji Kaneko, Yung-Che Tseng, Ritsuko Ohtani-Kaneko

**Affiliations:** 1 Department of Life Sciences, Toyo University, 1-1-1 Itakura, Oura, Gunma 374-0193 Japan; 2 Department of Aquatic Bioscience, Graduate School of Agricultural and Life Sciences, The University of Tokyo, 1-1-1 Yayoi, Bunkyo, Tokyo 113-8657 Japan; 3 Institute of Cellular and Organismic Biology, Academia Sinica, Nankang, Taipei City, 115 Taiwan, ROC

**Keywords:** Zoology, Endocrinology

## Abstract

The neuroplastic mechanism of sex reversal in the fish brain remains unclear due to the difficulty in identifying the key neurons involved. Mozambique tilapia show different reproductive behaviours between sexes; males build circular breeding nests while females hold and brood fertilized eggs in their mouth. In tilapia, gonadotropin-releasing hormone 3 (GnRH3) neurons, located in the terminal nerve, regulate male reproductive behaviour. Mature males have more GnRH3 neurons than mature females, and these neurons have been indicated to play a key role in the androgen-induced female-to-male sex reversal of the brain. We aimed to elucidate the signalling pathway involved in the androgen-induced increase in GnRH3 neurons in mature female tilapia. Applying inhibitors to organotypic cultures of brain slices, we showed that the insulin-like growth factor (IGF)-1 receptor (IGF-1R)/PI3K/AKT/mTOR pathway contributed to the androgen-induced increase in GnRH3 neurons. The involvement of IGF-1 and IGF-1R in 11-ketotestosterone (11-KT)-induced development of GnRH3 neurons was supported by an increase in *Igf-1* mRNA shortly after 11-KT treatment, the increase of GnRH3 neurons after IGF-1 treatment and the expression of IGF-1R in GnRH3 neurons. Our findings highlight the involvement of IGF-1 and its downstream signalling pathway in the sex reversal of the tilapia brain.

## Introduction

Sex reversal is a well-known phenomenon in teleost fish. Some teleosts naturally change their phenotypic sex during their lifetime, while others change it in response to environmental factors or treatments with hormones^1–3^. During sex reversal, sex change occurs not only in the gonads but also in the brain, which makes it possible to elicit the opposite sex-typical reproductive behaviours. The teleost brain and their gonads are known to be highly sexually labile throughout life^4–6^. However, the molecular and neuronal mechanisms of sex reversal in the teleost brain remain unclear.

There are some difficulties in elucidating the mechanism of sex reversal in teleost brains. First, male and female fish display the characteristic reproductive behaviours of their own species. When sex reversal occurs, reproductive behaviours change according to species-specific reproductive behaviours of the opposite sex. The species-specific difference in behavioural phenotypes could be mainly due to the evolutional divergence of a common mechanism including a molecular system and neural circuitry. Additionally, the developmental, genetic, physiological, and neuromodulatory mechanisms may also bias the evolution of behaviour, thereby affecting the evolvability of species-specific behaviour and the nervous system complexity. Second, sex reversal is induced in response to a variety of social, physical, environmental and hormonal stimuli. Thus, the regulatory mechanism related to sex reversal in the brain also depends on the type of key stimuli that induce it. These and other differences, such as the timing of sex reversal (e.g., during development or after maturation), make it difficult to identify the molecular and neuronal mechanisms of sex reversal in different species.

In this study, we investigated the regulatory mechanism of sex reversal in Mozambique tilapia (*Oreochromis mozambicus)*. Tilapias are important species in aquaculture. Clemens and Inslee produced all male populations of Mozambique tilapia larvae by adding 17α-methyltestosterone (MT), an artificial potent androgen, into the diet^7^. Since male tilapia grow approximately twice as fast as females, a monosex population of sex-reversed males is commercially produced using androgen-supplemented feed. The mechanisms underlying sex differentiation and hormone-induced sex reversal of the gonads during early development have been well studied in tilapia^8–13^. However, the mechanisms underlying sex reversal in the tilapia brain remain unsolved.

Female and male tilapia exhibit vastly different reproductive behaviours; males fight each other and build a large circular breeding nest, while females hold and brood fertilized eggs in their mouth (mouth brooding). Treatments with androgens, such as a fish natural androgen 11-ketotestosterone (11-KT), were reported to induce male-specific reproductive behaviours in most mature females within two weeks, while their gonads still contained numerous vitellogenic oocytes and the plasma concentration of 17β-estradiol (E2) remained high^14^. Thus, tilapia is a suitable model for investigating the regulatory mechanism of androgen-induced sex reversal in the brain.

Gonadotropin-releasing hormones (GnRHs) are neuropeptides responsible for reproduction in both vertebrates and non-vertebrates^4,15–17^. Advanced teleost fishes, including tilapias, have three GnRH subtypes (GnRH1, GnRH2, and GnRH3)^18–20^. It has been shown that GnRH neurons in the terminal nerve (TN) of dwarf gouramis play a modulatory role in male-specific reproductive behavior^21,22^. In addition, roles of TN-GnRH3 neurons have been demonstrated in modulation of mating preference for familiar males in medaka^23^. Using Nile tilapia (*Oreochromis niloticus*), Ogawa et al. have demonstrated that GnRH3 is a potent neuromodulator of male sexual behaviours such as nesting and aggression^24^. They injected antiserum against GnRH3 into the third ventricle of male Nile tilapia, which resulted in significantly decreased nest-building ability, nest size, and aggressive behavior^24^. Mature male Mozambique tilapia have been shown to have more GnRH3 neurons in the TN than females^14^. Treatment with 11-KT or MT, but not E2, increased the number of GnRH3 neurons in mature females to a level similar to that in males, and induced male-specific reproductive behaviors^14^. Furthermore, 11-KT has also been shown to promote neurogenesis in female tilapia and generate newly proliferated GnRH3 neurons^25^. The above-mentioned findings indicate the androgen-dependent regulation of GnRH3 neurons and their important role in male-specific reproductive behaviours in tilapia. However, the mechanism by which androgen affects GnRH3 neurons is yet to be understood.

We focused on GnRH3 neurons in Mozambique tilapia with respect to female-to-male sex reversal of the brain. The promoter of the *GnRH3* gene in Nile tilapia contains binding sites for many receptors, including the glucocorticoid receptor (GR) and thyroid hormone receptor (TR), but not for the androgen receptor (AR)^26^. Although the promotor gene sequences of GnRH3 in Mozambique tilapia remains still unknown, the genomic similarity of GnRH gene between these two tilapias is about 98% identity in BLAST. It is highly likely that Mozambique tilapia also lacks a binding site for AR in the promoter region of the GnRH3 gene. It is thus suggested that androgen binding to AR does not directly affect GnRH3 gene expressions. Instead, the effect of androgen on GnRH neurons is likely mediated through other molecules, such as hormones or neurotransmitters. In human and experimental mammals, it is well-known that the combined action of the somatotropic [growth hormone—insulin-like growth factor 1 (IGF-I)] and gonadotropic (GnRH-LH/FSH-sex steroid including androgen) hormone axes play important roles in the reproduction, the latter of which stimulates the former to produce the pubertal growth spurt^27^. In addition, the critical regulatory role of IGF-1 on various tissues including the hypothalamus has been indicated in the reproductive condition of vertebrates^28,29^. Due to the close relationship between IGF-1 and sex steroids in regards to reproductive functions, we examined the involvement of IGF-1 and its downstream pathway in the androgen-induced effects on GnRH3 neurons. This study is the first to propose a molecular mechanism by which GnRH3 neurons involved in male reproductive behaviours are positively regulated through the IGF-1 and IGF-1R / phosphoinositide 3-kinase (PI3K)/AKT/ mechanistic target of rapamycin (mTOR) signalling pathway during androgen-induced female-to-male sex reversal in the tilapia brain.

To examine the effects of androgen in the tilapia brain without any influence from other organs such as the gonads and liver, we used an organotypic culture of brain slices at the TN level, and pharmacologically investigated the possible involvement of IGF-1 signalling pathways.

## Results

### Effects of sex steroids on the number of GnRH3 neurons in cultured brain slices

**Figure 1:**
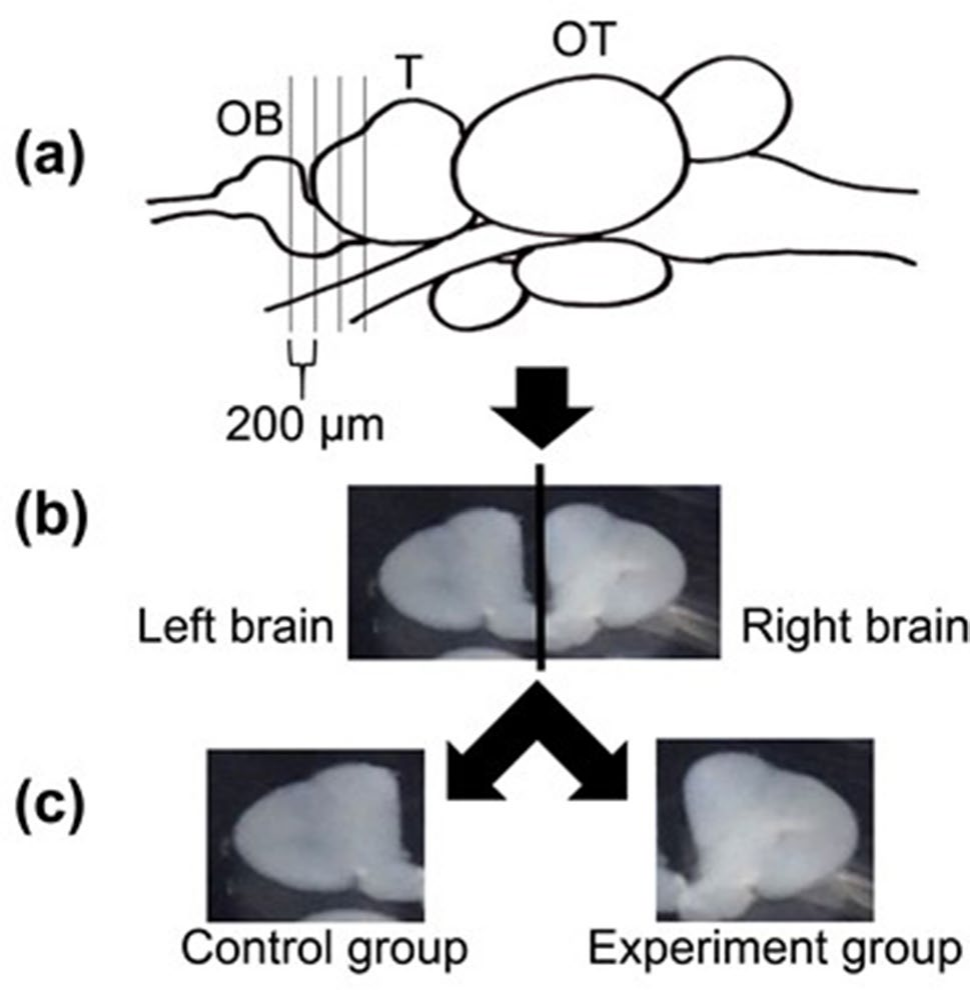
Brain slices of female tilapia. (**a**) A lateral view of the brain indicating the levels of the brain slices. (**b**) A cross-sectional slice of the brain cut at the terminal nerve. (**c**) Each brain slice was cut at the midline into right and left halves, which were used for the control and experimental groups, respectively. OB, olfactory bulb. T, telencephalon. OT, optic tectum.

**Figure 2:**
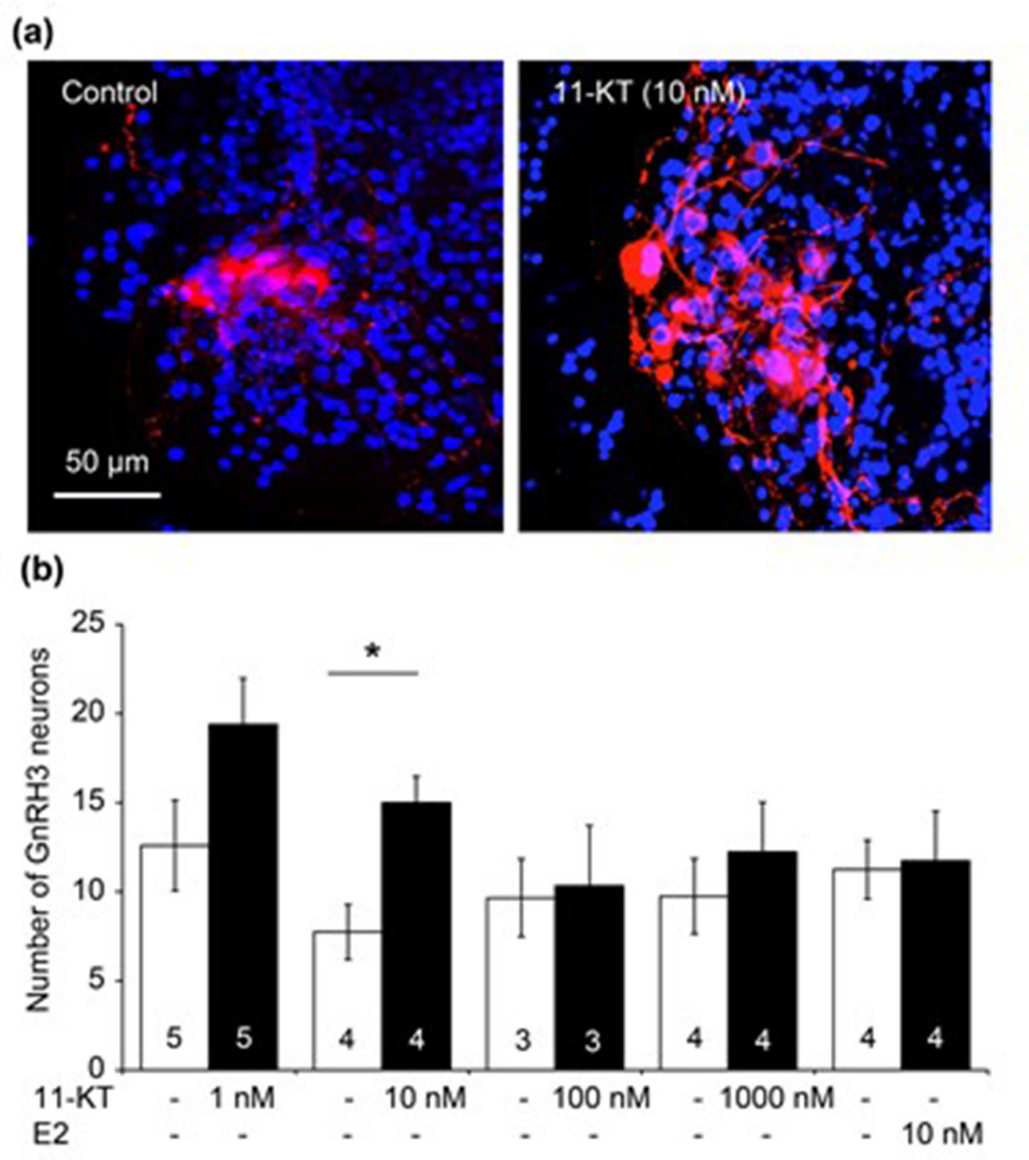
(**a**) Representative images of immunocytochemistry with gonadotropin-releasing hormone 3 (GnRH3) antibody. GnRH3 neurons (red) in control (left) and 11-ketotestosterone (11-KT)-treated (right) brain slices. Cell nuclei (blue) were stained with Hoechst 33342. (**b**) The numbers of GnRH neurons. White and black bars indicate the numbers of GnRH3 neurons in control slices and those treated with 11-KT (1, 10, 100 and 1000 nM ) and E2 (10 nM) (scale bar, 50 μm). An asterisk indicates a significant difference at *p*<0.05 (paired Student *t*-test). Numerals in bars indicate the numbers of animals used for preparing brain slices. Data are expressed as the mean ± standard error of the mean.

In order to examine whether 11-KT affects the number of GnRH3 neurons in vitro as previously observed in vivo^14,25^, we first examined the effect of 11-KT at different concentrations and that of 10 nM E2 on their numbers in female tilapia brain slices. Cross-sectional slices of the brain were cut at the TN level, and the slices were further cut into left and right halves (Fig. 1). One half of each brain slice was treated with either 11-KT or E2 (11-KT- or E2-treated group) for three days, while the other half was treated with the solvent alone (control group). GnRH3 neurons were immunostained with anti-GnRH3 (Fig. 2a), and the total number of GnRH3 neurons per animal was counted from all the half slices (2–4 slices) of the brain at the TN level originating from the respective animal (Fig. 2b). Then, the mean number of GnRH3 neurons was compared between the control, E2-treated and 11-KT-treated groups. Fig. 2b shows that 11-KT treatments with 1nM and 10 nM increased the number of immunostained GnRH3 neurons, and there was a statistically significant difference between the 10 nM 11-KT-treated and control groups (control *vs.* 11-KT (10 nM), *p* < 0.05, paired Student’s *t*-test). No increase was observed in the number of GnRH3 neurons in the 10 nM E2-treated group, compared to that in the control.

### Effects of IGF-1 on the number of GnRH3 neurons in cultured brain slices

**Figure 3:**
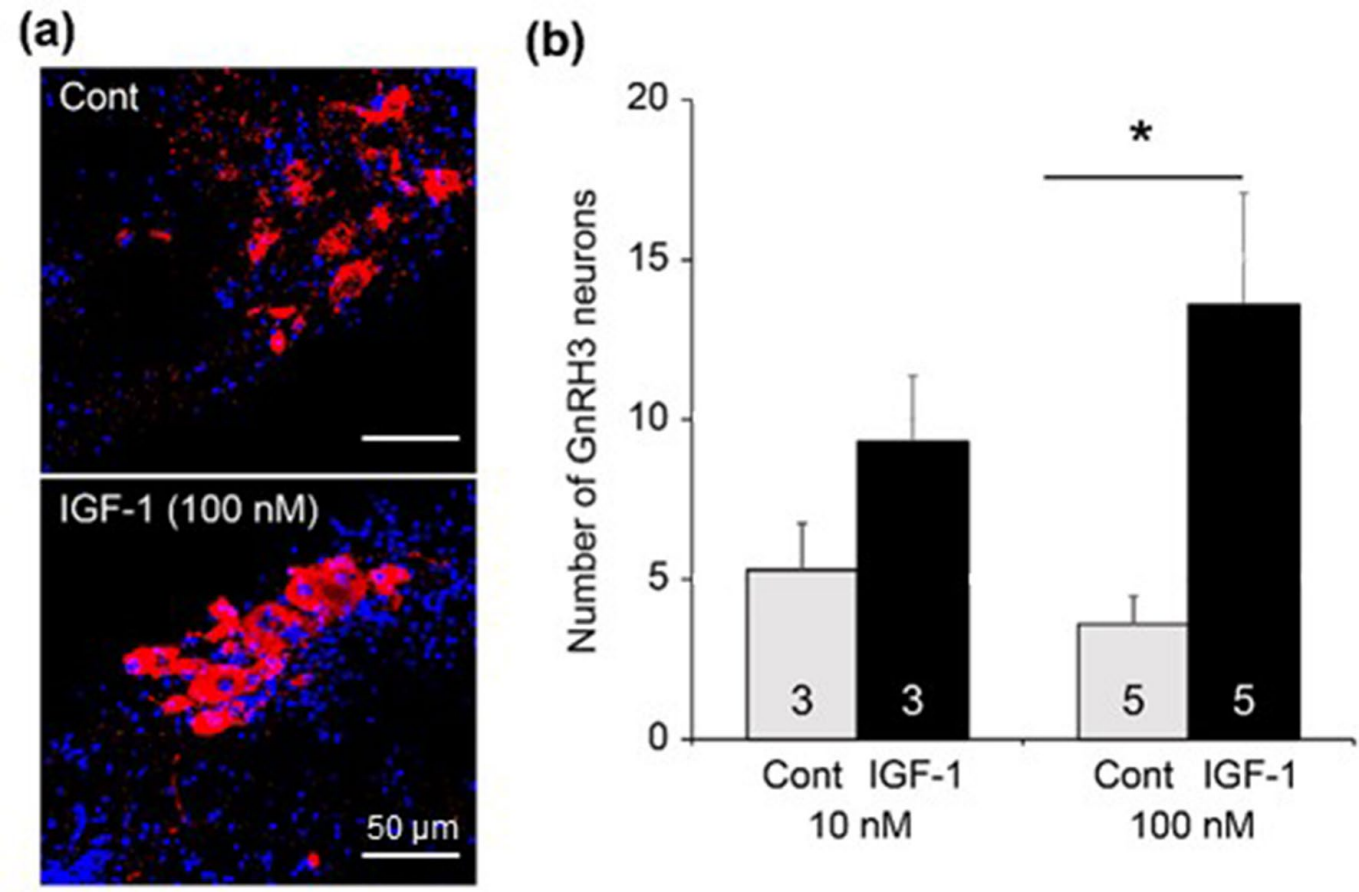
(**a**) Representative images of gonadotropin-releasing hormone 3 (GnRH3) neurons (red) in control (cont) and insulin-like growth factor 1 (IGF-1)-treated (100 nM) slices. Cell nuclei (blue) were stained with Hoechst 33342. (**b**) The numbers of GnRH3 neurons in control (Cont) and IGF-1 (10 nM and 100 nM)-treated slices (scale bar, 50 μm). An asterisk indicates a significant difference at *p*<0.05 (paired Student *t*-test). Numerals in bars indicate the numbers of animals used for preparing slices. Data are expressed as the mean ± standard error of the mean.

To examine the influence of IGF-1 on the number of GnRH3 neurons, the brain slices were treated with IGF-1 for three days. The number of GnRH3 neurons was compared between the control and IGF-1-treated slices originating from the same animal. Figure 3a, b show immunostained-GnRH3 neurons and the mean numbers of GnRH3 neurons per animal, respectively, in control and IGF-1-treated slices. Treatment with IGF-1 at 10 nM showed a tendency to increase the number of GnRH3 neurons compared to that of the controls. When 100 nM IGF-1 was added to the medium, the number of GnRH3 neurons was significantly greater than that in controls (control *vs.* 100 nM IGF-1, *p* < 0.05, paired Student’s *t*-test). These results revealed a stimulatory effect of IGF-1 on the number of GnRH3 neurons.

### Effects of 11-KT on IGF-1 mRNA expression

**Figure 4:**
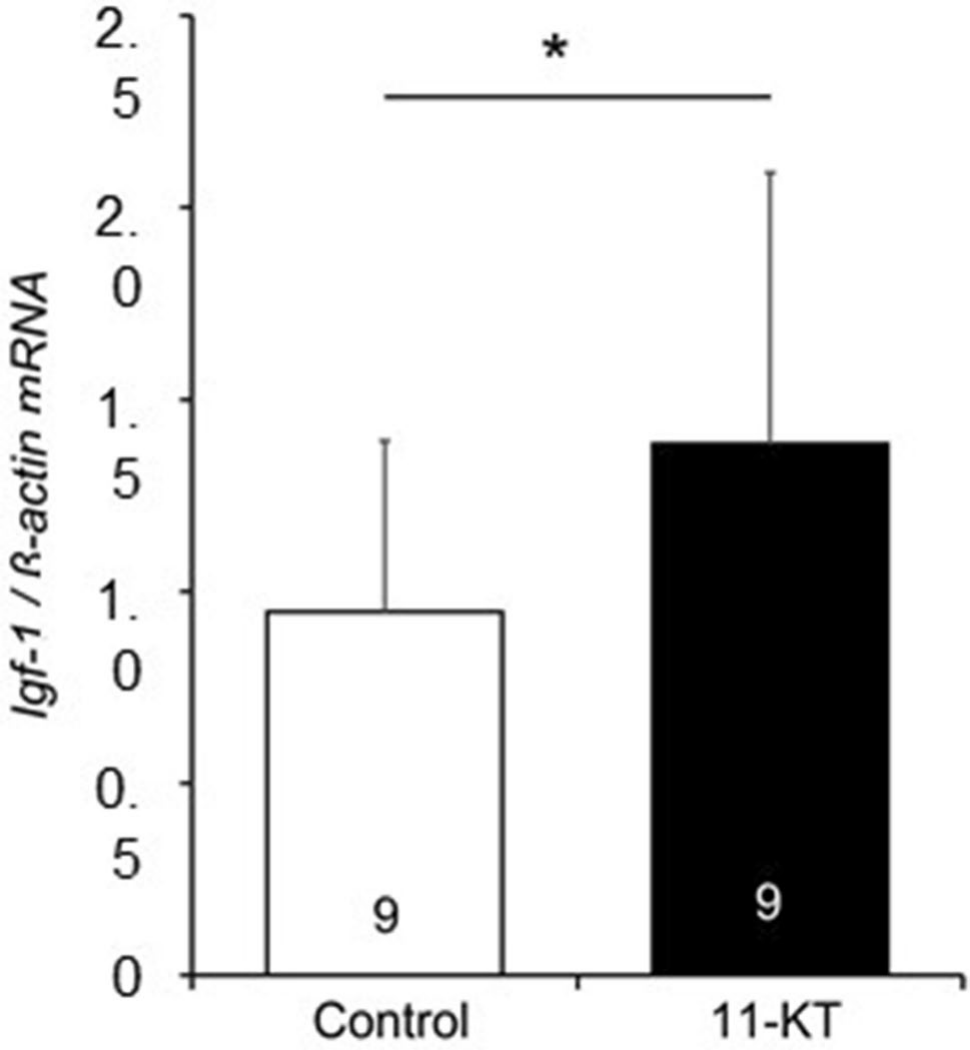
Changes in expressions of insulin-like growth factor 1 (*Igf-1)* mRNA in control and 11-ketotestosterone-treated slices 1 h after the treatment. An asterisk indicates a significant difference at *p *< 0.05 (paired Student’s *t*-test). Numerals in bars indicate the numbers of animals used for preparing slices. Data are expressed as the mean ± standard error of the mean.

*Igf-1* gene expression was examined in control and 11-KT groups using cultured halves of female brain slices originating from the same animal. The expression levels of *Igf-1* mRNA were measured 1 h after treatment with 10 nM 11-KT by real-time quantitative reverse transcription (qRT)-PCR, and compared between 11-KT-treated and control groups (Fig. 4). The expression of *Igf-1* mRNA was significantly higher in 11-KT-treated slices than in control slices (control *vs.* 11-KT, *p* < 0.05, paired Student’s *t*-test), indicating that 11-KT increased the expression of *Igf-1* mRNA in the brain.

### Effects of IGF-1R inhibitor on 11-KT-induced increase in GnRH3 neurons

**Figure 5:**
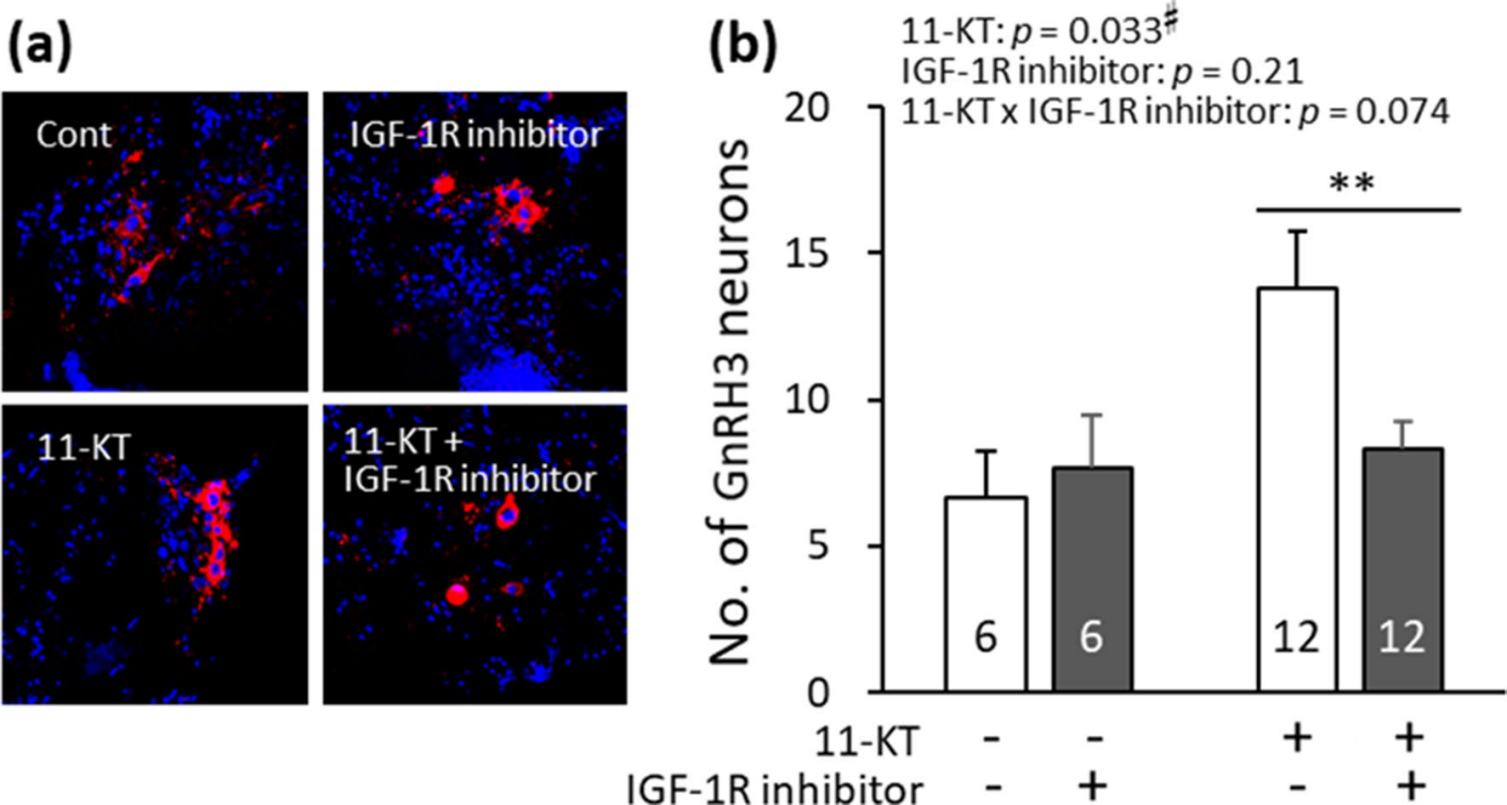
(**a**) Representative images of gonadotropin-releasing hormone 3 (GnRH3) neurons (red) in the following four groups of brain slices: control, insulin-like growth factor 1 receptor (IGF-1R) inhibitor (20 μM BMS-754807)-treated, 11-ketotestosterone (11-KT)-treated (10 nM), and 11-KT- (10 nM) and IGF-1R inhibitor (20 μM BMS-754807)-treated groups. Control and inhibitor-treated slices originated from the same animals, while 11-KT-treated slices and 11-KT- and inhibitor-treated slices originated from the same animals. Cell nuclei (blue) were stained with Hoechst 33342. (**b**) The numbers of GnRH3 neurons counted in the four groups of brain slices. Results from two-way analysis of variance (ANOVA) are shown in the top. Numerals in bars indicate the numbers of animals used for preparing slices. ^#^, a significant difference at *p* < 0.05 (two-way ANOVA). **, a significant difference at *p *< 0.005 [paired *t*-test, Bonferroni Correction (α new = α original / n = 0.01/2]. Data are expressed as the mean ± standard error of the mean.

To examine the contribution of IGF-1R to 11-KT-induced increase in GnRH3 neurons, effects of IGF-1R inhibitor were studied. Micrographs in Fig. 5a show immunostained-GnRH3 neurons (red) in four brain slice groups: control, IGF-1R inhibitor (BMS-754807, 20 μM)-treated, 11-KT-treated, 11-KT- and IGF-1R inhibitor-treated groups. Control and inhibitor-treated animals originated from the same animal, as shown in Fig. 1. Similarly, 11-KT-treated and 11-KT- and IGF-1R inhibitor-treated groups originated from the same animal. The graphs in Fig. 5b indicate the average number of GnRH3 neurons per animal in each of the four groups.

As shown in Fig. 5b, two-way ANOVA revealed a significant effect of 11-KT (*p* = 0.033) but no significant effect of IGF-1R inhibitor (*p* = 0.21). There was no significant interaction between 11-KT and IGF-1R inhibitor (*p* = 0.074). Because a two-way ANOVA and commonly used post-hoc tests such as the Tukey test are not suitable for the comparison of individuals with the correspondence, we thus used a paired *t*-test for the statistical analysis of the numbers of GnRH3 neurons in the half brain slices originating from the same animal. Furthermore, in using Bonferroni correction to avoid the probability of committing a type I error, *p* < 0.025 was considered statistically significant for paired *t*-test. The difference in the number of GnRH3 neurons between the ‘11-KT’ and ’11-KT & IGF-IR inhibitor’ groups was significant (*p* = 0.0043, paired *t*-test). The difference in the number of GnRH3 neurons between the ‘control’ and ’IGF-IR inhibitor’ groups was not significant (*p* = 0.57, paired *t*-test). That is, IGF-1R inhibitor significantly suppressed the 11-KT-induced increase in GnRH3 neurons.

### IGF-1R-immunoreactivities in GnRH3 neurons

**Figure 6:**
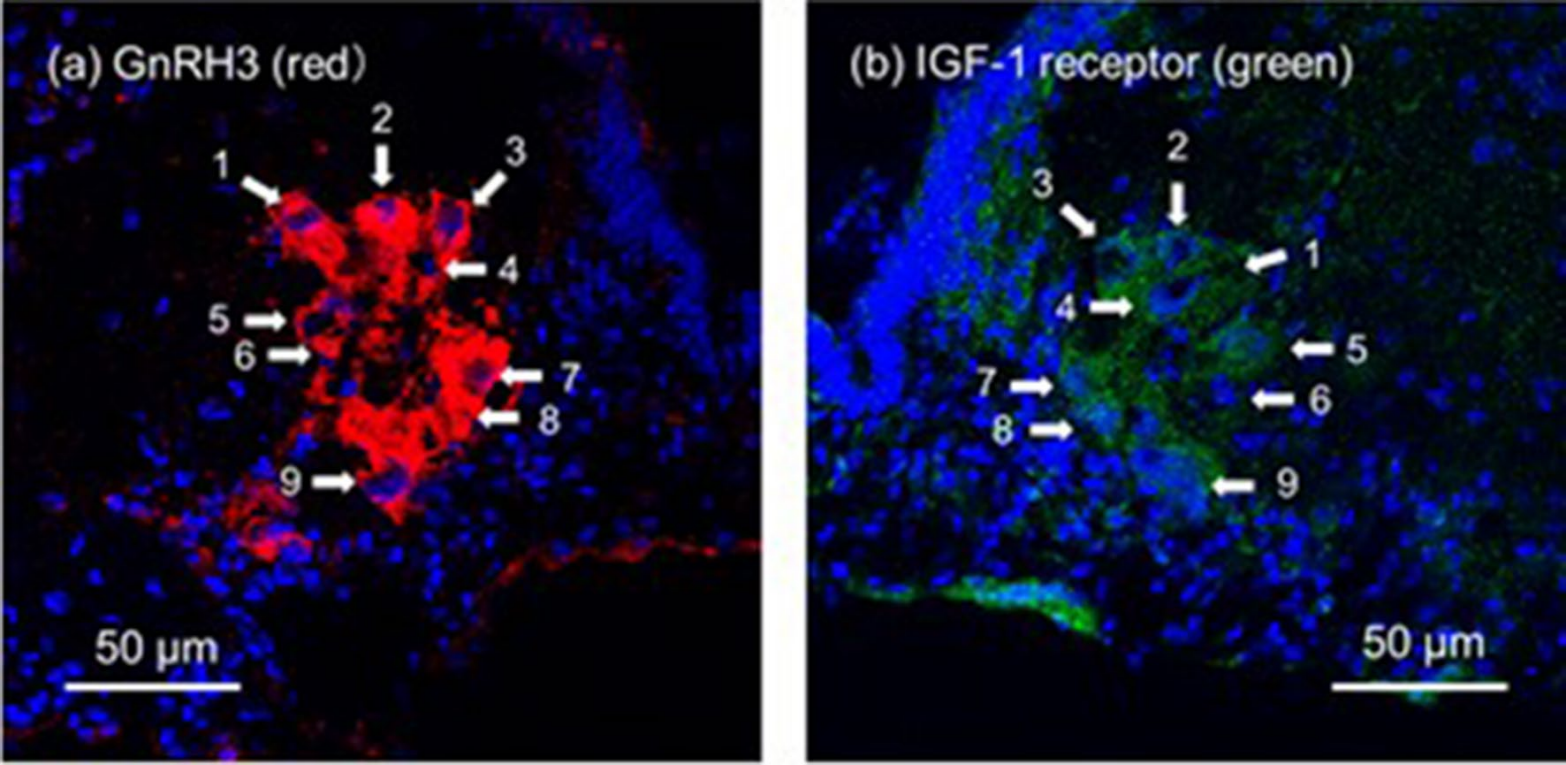
Representative images of gonadotropin-releasing hormone 3 (GnRH) neurons [red in (a)] and insulin-like growth factor 1 receptor immunoreactivities [green in (b)] on a pair of mirror-image frozen sections from 11-ketotestosterone-injected females. The same numerals in respective pictures indicate the same cells appearing on the two sections. Cell nuclei (blue) were stained with Hoechst 33342.

To investigate whether GnRH3 neurons in 11-KT-treated females express IGF-1R, a pair of adjacent frozen sections with mirror images were subjected to immunocytochemical staining with rabbit polyclonal antibodies against GnRH3 and IGF-1R. GnRH-neurons (red in Fig. 6a) showed IGR-1R immunoreactivity (green in Fig. 6b), although IGF-1R immunoreactivity was also observed in the cells without GnRH3-immunoreactivities near the ventricle.

### Effects of PI3K/AKT/mTOR pathway inhibitors on 11-KT-induced increase in the number of GnRH3 neurons

**Figure 7:**
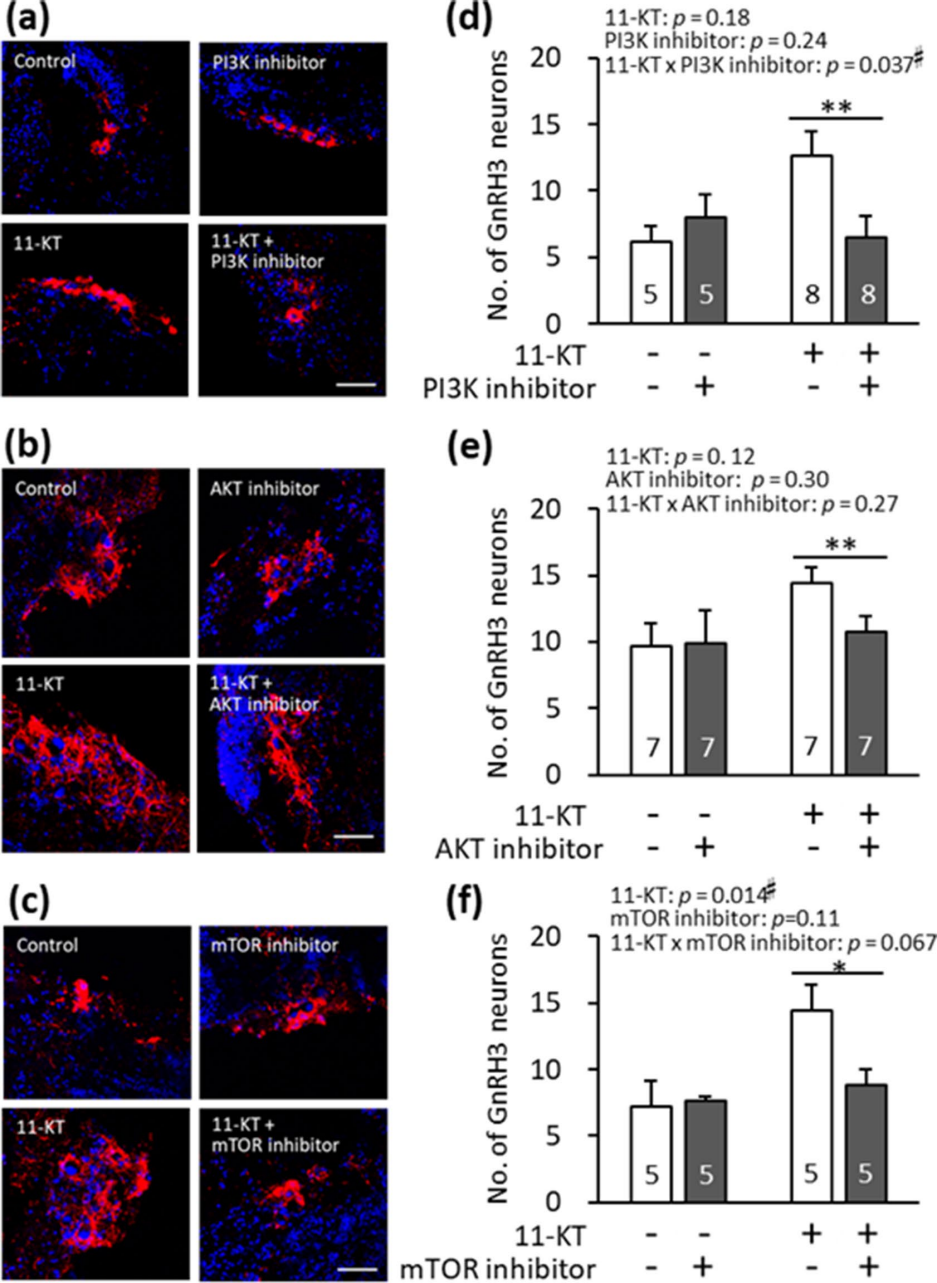
(**a**)–(**c**) Representative images of gonadotropin-releasing hormone 3 (GnRH3) neurons (red) in the following four groups of brain slices: control, inhibitor-treated, 11-ketotestosterone (11-KT)-treated (10 nM), and 11-KT- (10 nM) and inhibitor-treated groups. Cell nuclei (blue) were stained with Hoechst 33342. (**d**)–(**f**) The numbers of GnRH3 neurons in the four groups. Control and inhibitor-treated slices originated from the same animals, while 11-KT-treated slices and 11-KT and inhibitor-treated slices originated from the same animals. Inhibitors used in (**a**)–(**c**) and (**d**)–(**f**) were as follows: (**a**, **d**) phosphoinositide 3-kinase (PI3K) inhibitor (LY294002), (b, e) AKT inhibitor (GDC-0068), (**c**, **f**) mechanistic target of rapamycin (mTOR) inhibitor (rapamycin). Results from two-way analysis of variance (ANOVA) are shown in the top. Numerals in bars indicate the numbers of animals used for preparing slices. ^#^, a significant difference at *p* < 0.05 (two-way ANOVA). * and **, a significant difference at *p *< 0.025 and *p *< 0.005, respectively [paired *t*-test, Bonferroni Correction (α new = α original / n = 0.05/2 and 0.01/2]. Data are expressed as the mean ± standard error of the mean.

To examine the signalling pathway involved in the androgen-induced increase of GnRH3 neurons, one of the inhibitors examined was added to the medium simultaneously with 10 nM 11-KT, and their suppressive effect on the 11-KT-induced increase of GnRH3 neurons was examined by counting the number of immunostained GnRH3 neurons in the brain slices. The following inhibitors were examined in this study: PI3K inhibitor (LY294002), AKT inhibitor (GDC-0068), and mTOR inhibitor (rapamycin). Micrographs in Fig. 7a–c show immunostained-GnRH3 neurons (red) in four brain slice groups: control, inhibitor-treated, 11-KT-treated, and 11-KT- and inhibitor-treated groups. Control and inhibitor-treated brain slices originated from the same animals. Similarly, 11-KT-treated and 11-KT- and inhibitor-treated brain slices originated from the same animal. Graphs in Fig. 7d–f indicate the average numbers of GnRH3 neurons per animal in the four groups.

The effect of PI3K inhibitor (LY294002) was examined (Fig. 7a, d). A two-way ANOVA showed no significant effect of 11-KT (*p* = 0.18) and PI3K inhibitor (*p* = 0.24), but a significant 11-KT × PI3K inhibitor interaction (*p* = 0.037). The differences among four groups were not significant by a two-way ANOVA followed by Tukey post-hoc test. We next used a paired *t*-test for the statistical analysis of the numbers of GnRH3 neurons in the half brain slices originating from the same animal. Paired *t*-test showed that the difference in the number of GnRH3 neurons between the ‘11-KT’ and ’11-KT & PI3K inhibitor’ groups was significant (*p* = 0.0029), but not significant between ‘control’ and ‘PI3K inhibitor’ groups (*p* = 0.19). That is, PI3K inhibitor significantly suppressed the 11-KT-induced increase in GnRH3 neurons.

Treatment with the AKT inhibitor (GDC-0068, pan-AKT inhibitor targeting AKT1, AKT2, and AKT3) was first examined at different concentrations (1, 10, and 20 μM). GDC-0068 at 10 and 20 μM significantly reduced the 11-KT-induced increase in the number of GnRH3 neurons. The effects of GDC-0068 at 20 μM were then examined in four brain slice groups (Fig. 7b and e). A two-way ANOVA showed no significant effect of 11-KT (*p* = 0.12), AKT inhibitor (*p* = 0.30), or 11-KT × AKT inhibitor interaction (*p* = 0.27). While AKT inhibitor alone did not affect the number of GnRH3 neurons (‘control’ *vs* ‘AKT inhibitor’ groups, *p* = 0.95, paired *t*-test), AKT inhibitor significantly suppressed the 11-KT-induced increase in the number of GnRH3 neurons (*p* = 0.0037, paired *t*-test) (Fig. 7e).

The effect of the mTOR inhibitor, rapamycin, (10 μM) was examined (Fig. 7c and f). A two-way ANOVA revealed a significant effect of 11-KT (*p* = 0.014) but no significant effect of mTOR inhibitor (*p* = 0.11). There was no significant interaction between 11-KT and mTOR inhibitor (*p* = 0.067). The effect of 11-KT on the number of GnRH3 neurons was confirmed. Paired t-test showed that the treatment with mTOR inhibitor alone did not affect the number of GnRH3 neurons (‘control’ *vs* ‘mTOR inhibitor’ groups, *p* = 0.85), but the treatment of mTOR inhibitor significantly suppressed the 11-KT-induced increase in the number of GnRH3 neurons (‘11-KT’ *vs.* ‘11-KT & mTOR inhibitor’ groups, *p* = 0.017) (Fig. 7f).

### Effects of ERK and JAK inhibitors on 11-KT-induced increase in GnRH3 neurons

**Figure 8:**
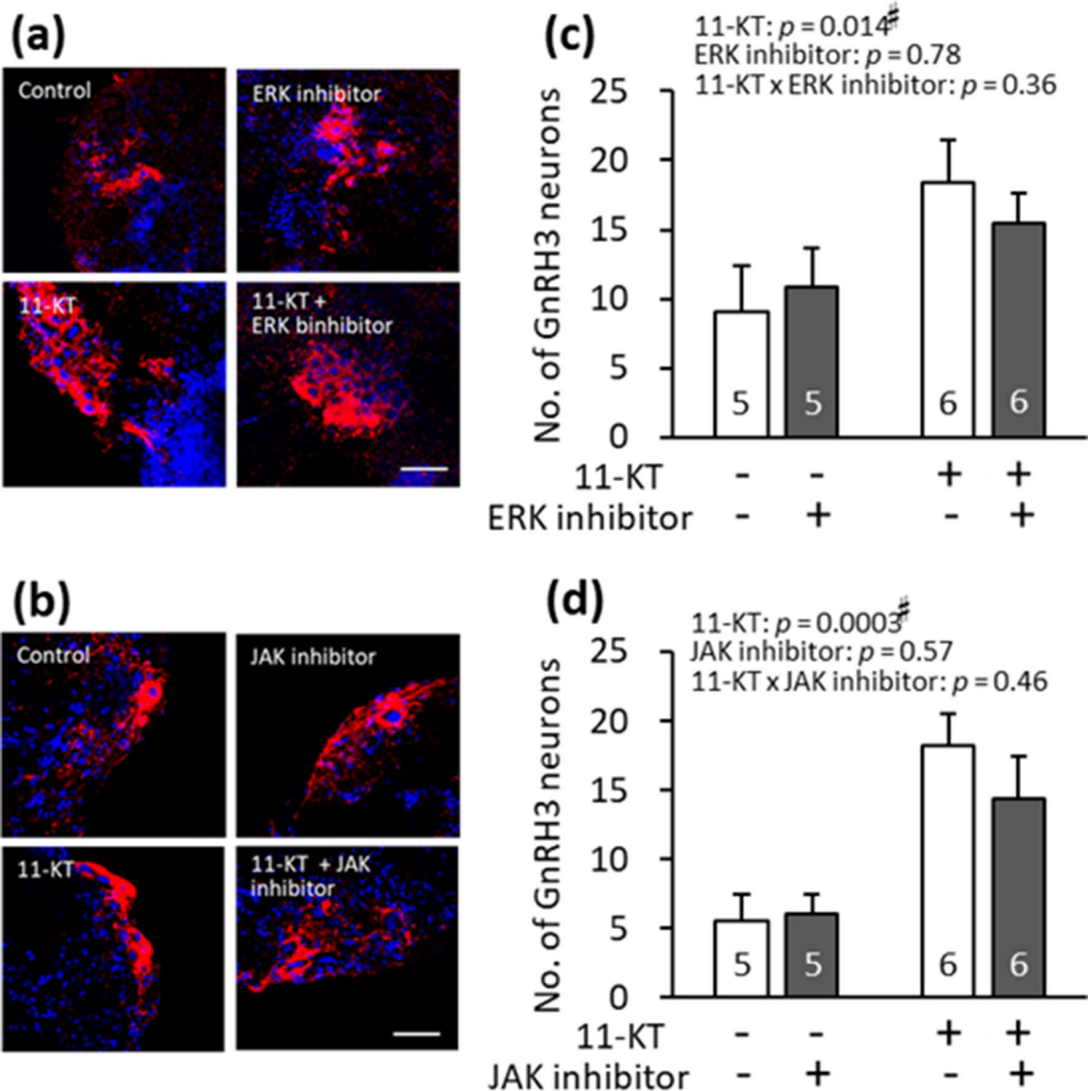
(**a**, **b**) Representative images of gonadotropin-releasing hormone 3 (GnRH3) neurons (red) in the following four groups of brain slices: control, inhibitor-treated, 11-ketotestosterone (11-KT)-treated (10 nM), and 11-KT- (10 nM) and inhibitor-treated groups. Cell nuclei (blue) were stained with Hoechst 33342. (**c**, **d**) The numbers of GnRH3 neurons in the four groups. Control and inhibitor-treated slices originated from the same animals, while 11-KT-treated slices and 11-KT- and inhibitor-treated slices originated from the same animals. Inhibitors used in (**a**, **c**) and (**b**, **d**) were as follows: (**a**, **c**) ERK inhibitor (20 μM U0126), (**b**, **d**) JAK inhibitor (100 nM, InSolution^TM^ JAK inhibitor 1). Results from two-way analysis of variance (ANOVA) are shown in the top. Numerals in bars indicate the numbers of animals used for preparing slices. ^#^, a significant difference at *p* < 0.05 (two-way ANOVA). Data are expressed as the mean ± standard error of the mean.

The effects of the extracellular signal-regulated kinase (ERK) inhibitor (U0126) and Janus kinase (JAK) inhibitor (InSolution^TM^ JAK inhibitor 1) were similarly examined (Fig. 8). Micrographs in Fig. 8a, b show immunostained-GnRH3 neurons (red) in four brain slice groups: control, inhibitor-treated, 11-KT-treated, and 11-KT- and inhibitor-treated groups. Control and inhibitor-treated brain slices originated from the same animals. Similarly, 11-KT-treated and 11-KT- and inhibitor-treated brain slices originated from the same animal.

As for ERK inhibitor, two-way ANOVA revealed a significant effect of 11-KT (*p* = 0.014) but no significant effect of ERK inhibitor (*p* = 0.78). There was no significant interaction between 11-KT and ERK inhibitor (*p* = 0.36) (Fig. 8c). In addition, paired *t*-test showed no significant differences in the number of GnRH3 neurons between ‘11-KT’ and ’11-KT & ERK inhibitor’ groups (*p* = 0.093) or between ‘control’ and ‘ERK inhibitor’ groups (*p* = 0.56) (Fig. 8c).

As for JAK inhibitor, two-way ANOVA revealed a significant effect of 11-KT (*p* = 0.0003) but no significant effect of JAK inhibitor, InSolution^TM^ JAK inhibitor I, (100 nM) (*p* = 0.57). No significant interaction was seen between 11-KT and JAK inhibitor (*p* = 0.46). In addition, paired *t*-test showed no significant differences in the numbers of GnRH3 neurons between ‘11-KT’ and ’11-KT & JAK inhibitor’ groups (*p* = 0.14) or between ‘control’ and ‘JAK inhibitor’ groups (*p* = 0.65) (Fig. 8c).

Taken together, we showed that the inhibitors of IGF-1R, PI3K, AKT, and mTOR were highly effective in suppressing the 11-KT-induced increase of GnRH3 neurons in brain slices originating from mature females. Contrastingly, inhibitors of ERK and JAK did not significantly suppress the effect of 11-KT on the number of GnRH3 neurons.

## Discussion

### Role of IGF-1 and IGF-1R/PI3K/AKT/mTOR signalling pathway with respect to the effects of 11-KT on GnRH3 neurons in the female tilapia brain

According to the present results obtained with the TN-containing brain slices of mature females (Fig. 2), the *in vitro* treatment with 11-KT increased the number of GnRH3 neurons, indicating that 11-KT can increase the number of GnRH3 neurons without mediation via other organs, such as the gonad and liver. Since there are no AR binding sites in the promoter regions of GnRH3 gene in tilapia^26^, we considered that IGF-1 might mediate the action of 11-KT on GnRH3 neurons. Our hypothesis is strongly supported by the following four findings: (1) Treatment with IGF-1 increased the number of GnRH3 neurons (Fig. 3). (2) The expression of IGF-1 mRNA increased shortly after 11-KT treatment (Fig. 4). (3) The potent IGF-1R inhibitor, BMS-754807, strongly suppressed the 11-KT-induced increase of GnRH3 neurons (Fig. 5). (4) Immunohistochemical staining with anti-GnRH3 and anti-IGF-1R antibodies revealed the co-localization of IGF-1R and GnRH3 (Fig. 6). These results support the possibility that the effect of 11-KT on GnRH3 neurons is mediated by IGF-1 produced in the brain and IGF-1R on GnRH3 neurons. Furthermore, studies using inhibitors (Figs. 5, 7, and 8) clearly indicate the contribution of the IGF-1R downstream signalling pathway, PI3K/AKT/mTOR, to the effect of 11-KT on GnRH3 neurons. Therefore, our findings indicate that the effect of 11-KT on the number of GnRH3 neurons is mediated by the IGF-1 and IGF-1R/PI3K/AKT/mTOR signalling pathways.

Although there were no binding sites for ARs in GnRH3 promoters^26^, the expression of AR mRNA was reported near, but not in, the TN of female medaka^30^. Ogawa and Parhar showed by single-cell gene profiling that, although *Arβ* mRNA as well as *Tr*, *Gr*, and *Er* mRNAs were expressed in GnRH3 neurons, the expression level of *Arβ* mRNA was so low that nested-PCR was necessary to observe it^31^. Combining these results with ours, it can be proposed that the effect of 11-KT on GnRH3 neurons is mediated by other molecules such as IGF-1 and its receptor, although it cannot be completely ruled out that 11-KT affects GnRH3 neurons directly.

### The role of IGF-1 and IGF-1R/PI3K/AKT/mTOR signalling pathway in the regulatory process of GnRH neurons reported in other animals

IGF-1 is known to possess powerful neuroprotective effects in promoting neuronal survival, neuronal differentiation, neurite elongation, neurogenesis, and neurite regeneration *in vivo* and *in vitro*^32^. It is a regulator of reproductive neuroendocrine function that affects the development and functions of GnRH neurons in the vertebrate brain, although this has been reported mainly in mammals^33,34^. GnRH perikarya express IGF-1R before puberty in mice, suggesting a potential direct anatomical locus where IGF-1 can control reproductive development and function^33,35^. Furthermore, the action of IGF-1 on IGF-1R in GnRH neurons regulates the onset of puberty in rodents^36,37^.

In fish like Mozambique tilapia, while IGF-1 and IGF-2 are mainly produced in the liver, their transcripts are expressed in various tissues including the brain^38^. The absolute amounts of *Igf-1* and *Igf-2* mRNAs were also determined in many regions, such as the liver and brain of Nile tilapia^39^. In yellow catfish, the expression of *Igf-1* mRNA in the hypothalamus was higher in adult males than in adult females^40^. Additionally, the effect of androgen (MT) treatment affected its expression in a sex-dependent manner; MT treatment in females increased the expression of *Igf-1* mRNA.

The activation of IGF-1R leads to the stimulation of the following two major downstream signalling pathways: (i) the PI3K-AKT-mTOR pathway and (ii) Ras-Raf-MAP pathway^29^. Regarding the relationship between GnRH and the intracellular signalling pathway following IGF-1R, Shao et al. investigated the expression of genes and proteins related to the IGF-1 pathway in the rat hypothalamus after combined treatments with di-(2-ethylhexyl) phthalate (DEHP), a common environmental endocrine disruptor^41^. Their study indicated that DEHP might activate the hypothalamic GnRH neurons prematurely through the IGF-1 and IGF-1R/PI3K/AKT/mTOR signalling pathway to promote GnRH release. It was recently reported that administering IGF-1 significantly increased the firing rate and frequency of spontaneous postsynaptic currents in rodents, and that IGF-1R and PI3K are involved in this process^42^. These findings indicate that IGF-1 affects the activity of GnRH neurons through IGF-1R and the PI3K/AKT/mTOR pathways in rodents.

In teleosts, there are only a few studies on IGF-1R downstream signalling pathways in GnRH neurons. Onuma et al. revealed that IGF-1 affects the development of GnRH2 and GnRH3 through IGF-1R in zebrafish embryos^43^. They also examined whether PI3K inhibitors and/or ERK inhibitors disturbed the distribution of GnRH3 neurons in embryos. Treatment of embryos with a PI3K inhibitor (LY294002 or wortmannin) caused abnormal GnRH3 distribution similar to that in IGF signalling-deficient embryos. Contrastingly, U0126 had no effects. These results indicate that the influence of IGF-1 on the development and migration of GnRH2 and GnRH3 neurons was mediated through IGF-1R and the subsequent PI3K signalling pathway. According to findings regarding GnRH neurons in mammals and teleosts, it is highly likely that IGF-1 and the IGF-1R/PI3K/AKT/mTOR pathway play an important regulatory role in GnRH3 neurons in Mozambique tilapia.

In our previous studies, when 11-KT induced male-specific reproductive behaviours in mature female tilapia, GnRH3 neurons were increased in the TN region^14,25^. Moreover, newly proliferated GnRH3 neurons have been found in the TN of 11-KT-treated mature females^25^. Considering these findings with the present results, we conclude that IGF-1 and the IGF-1R/PI3K/AKT/mTOR signalling pathway are likely involved in the 11-KT-induced sex reversal of the female brain in terms of GnRH3 neurons.

### Reduced involvement of ERK and JAK in the 11-KT-induced increase of GnRH3 neurons

IGF-1R signalling can also activate the Ras/Raf/Mek/ERK pathway, which is broadly involved in the regulation of neuron proliferation, differentiation, and apoptosis^43–45^. The co-activation of the ERK and AKT signalling pathways has been reported to promote the survival of Gn10 GnRH cells or GnRH neurons^46,47^. Additionally, positive regulation of *Gnrh* mRNA levels induced by kisspeptin was mediated through the activation of ERK and AKT signalling pathways in mammalian GT1-7 (a clonal cell line of mature, differentiated GnRH neurons) and GN11 (a clonal cell line with GnRH expression) cells^48^. JAK2 is necessary for the neuroendocrine control of female reproduction and development of GnRH neurons^49,50^. *In vitro*, GnRH neurons and GnRH cell lines respond to a variety of ligands, including cytokines, which activate the JAK/signal transducers and activators of transcription intracellular signalling pathway^51,52^. However, in this study, the ERK and JAK inhibitors did not significantly suppress the 11-KT-induced increase of GnRH3 neurons, indicating a reduced contribution of ERK and JAK in mediating the effect of 11-KT on GnRH3 neurons compared to the PI3K/AKT/mTOR pathway.

We focused on GnRH3 neurons, which have been reported to control male-specific reproductive behaviour in tilapia^24^, and found a possible role of IGF-1 in mediating the effect of androgen on GnRH3 neurons. IGF-1 is a very important molecule commonly used in controlling reproduction by regulating GnRH neurons in mammals, as well as in controlling somatic development in vertebrates. Therefore, our findings propose an attractive idea that IGF-1 and its downstream signalling pathway play an important role during the sex reversal of the brain in tilapia.

## Materials and methods

### Animals

All experimental protocols used in this study on fish were approved by the Institutional Animal Care and Use Committee of Toyo University Itakura Campus and all experiments were performed in accordance with relevant guidelines and regulations. Furthermore, the present study were performed in accordance with ARRIVE guidelines (Animal Research: Reporting *in Vivo* Experiments). Female Mozambique tilapia (total length, 7–10 cm; body weight, 12–20 g) were maintained in fresh water at 25 °C and used for the experiments.

### Brain slice cultures

#### Preparation of brain slices

Brain slices at the level of the TN were prepared according to the protocol provided by Dr. Satoshi Ogawa in Prof. Ishwar S. Parhar’s laboratory (Monash University, Malaysia). Mature female tilapia fish were deeply anaesthetized with 0.2% 3-aminobenzoic acid ethyl ester (MS222, Sigma-Aldrich, St. Louis, MO, USA) and decapitated. The brain was removed and stored in cooled Ringer’s saline containing 124 mM NaCl, 3 mM KCl, 2 mM CaCl_2_, 2 mM MgSO_4_, 1.25 mM NaH_2_PO_4_, 26 mM NaHCO_3_, and 10 mM glucose (pH 7.35). The brain area from the olfactory bulb to the middle of the telencephalon was dissected and embedded in 4% low melting point agarose (Sigma-Aldrich) within embedding moulds. The embedding moulds were then placed on ice. After the agarose hardened (~5 min), the embedded brain and agarose were glued to the stage of a vibratome (Dosaka-EM Co., Ltd, Kyoto, Japan) and cut into 200-μm cross-sectional slices. The slices that covered all the GnRH neurons in the TN were collected in a dish containing Wash Medium [DMEM phenol free (Thermo Fisher Scientific, Waltham, MA, USA)] supplemented with 134 U/ml penicillin (Cosmo Bio Co., Ltd, Tokyo, Japan), 0.25 glutamate (Sigma-Aldrich), 1 × B-27 serum-free (Thermo Fisher Scientific), 0.13 mM streptomycin (MeijiSeika Pharma, Tokyo, Japan), and 0.5 L-glutamine (Sigma-Aldrich)]. Each slice was then divided into equal halves by cutting at the midline (Fig. 1), and the halves were separately transferred to wells of a sterilised 96-well culture plate (Corning Inc., Corning, NY, USA) containing 200 μl of culture medium (CM) [Leibovitz’s 15 (L-15) medium (Sigma-Aldrich) supplemented with 100 U/ml penicillin, 0.1 mg/ml streptomycin, 10 mM HEPES buffer (Sigma-Aldrich), and 10 mM D-glucose (Wako Pure Chemical Co., Osaka, Japan)]. The plates were then incubated at 25 °C.

The CM was changed every 24 h. After brain slices were cultured for 72 h, they were fixed with 4% paraformaldehyde solution in phosphate buffered saline (PBS) for 1 h for immunocytochemistry.

### Treatment of brain slices with11-KT or E2

First, 10 mM 11-KT or E2 was prepared in 99.5 % ethanol (Wako Pure Chemical Co.). In the experimental groups, after 11-KT and E2 were diluted with CM, they were added to a 96-well plate at final concentrations of 1, 10, 100, and 1000 nM for 11-KT and 10 nM for E2. For the control groups, ethanol was added at the same concentration as that of the corresponding experimental group. The slices at the TN were cut into left and right halves (Fig. 1). One half of the brain slice was treated with either 11-KT or E2 (11-KT or E2 group) and the other half was treated with ethanol (control group). We used 3–5 animals for the respective concentrations of 11-KT and four animals for 10 nM E2.

### Treatment of brain slices with IGF-1

A solution of 50 μM IGF-1 (IGF-1 Gilthead Seabream, PROSPEC, Hamada, Israel) was prepared using CM. Then, IGF-1 was added to a 96-well plate at a final concentration of 10 or 100 nM. For the control group, an equal volume of CM was added to each well. Slices at the TN were cut into left and right halves, which were used for the IGF-1 and control groups, respectively. Thus, IGF-1-treated and control slices originated from the same fish. We used slices from 3 to 5 animals for the respective concentrations of IGF-1.

### Treatment of brain slices with 11-KT and/or one of the inhibitors

The following inhibitors were used in this study: IGF-1R inhibitor (BMS-754807; Funakoshi Co. Ltd., Tokyo, Japan), PI3K inhibitor (LY294002; Wako Pure Chemical Co.), AKT inhibitor (GDC-0068; Funakoshi Co. Ltd.), mTOR inhibitor (Rapamycin, Funakoshi Co. Ltd.), ERK inhibitor (U0126; Cosmo Bio Co. Ltd.), and JAK inhibitor (InSolution^TM^ JAK inhibitor I, Calbiochem, San Diego, CA, USA).

The right and left halves of brain slices were used. In ‘the control group’, the slice was incubated in the CM containing solvent (ethanol, DMSO, or saline) at the same concentration as that used to dissolve the respective inhibitor and 11-KT. In ‘the inhibitor group’, the slice was incubated in CM containing one of the inhibitors and the solvent to dissolve 11-KT. In ‘the 11-KT group’, the slice was incubated with 10 nM 11-KT, and the solvent was added at the same concentration as that used to dissolve the respective inhibitor. In ‘the 11-KT + inhibitor group’, the slices were incubated in CM containing 10 nM 11-KT and one of the inhibitors.

The slices for ‘control’ and ‘inhibitor’ groups originated from the same animal. Similarly, the slices for ‘11-KT’ and ‘11-KT + inhibitor’ groups originated from the same animal. We used 10–14 animals for the two pairs of the following four groups: the ‘control’, ‘inhibitor’, ‘11-KT’, and ‘11-KT- and inhibitor’ groups.

### Immunostaining with anti-GnRH3 antibodies

The brain slices at 72 h after cultivation were fixed with 4% paraformaldehyde. They were washed gently with PBS three times. Immunocytochemistry with an anti-GnRH3 antibody was performed using a custom-made rabbit anti-GnRH3 antibody (×7000, produced by Protein Purify, Isesaki, Japan) and Alexa 555-labelled goat anti-rabbit IgG (×500, Thermo Fisher Scientific, Ltd.). For nuclear staining, Hoechst 33342 solution (1000×, Wako Pure Chemical Co.) was used. After staining, coverslips were mounted with ProLong Gold antifade reagent (Thermo Fisher Scientific, Ltd.). The stained slices were observed using a confocal laser microscope (LSM 5 PASCAL, Carl-Zeiss AG, Jena, Germany). Since GnRH3 neurons are located so close to each other that it is difficult to count them accurately on a single sectional image. Then, we obtained a series of images in the z-stack and merged them to count the actual number of GnRH3 neurons in each slice. The total number of GnRH3 neurons per animal was calculated by summing the number of GnRH3 neurons on each half side of the brain slices (2–4 slices) originating from the same animal.

### Immunostaining with anti-IGF-1R and GnRH3 antibodies

Sexually mature female tilapia of approximately equal size (n=3, approximately 30 g in body weight and 15 cm in total length) were used. 11-KT was dissolved in sesame oil at a concentration of 1.0 mg/ml. Three mature females were intraperitoneally injected with 11-KT at 5 μg/g body weight (11-KT-treated females). After the injections, the fish were maintained individually in 50-L glass tanks. Two days after the injection, they were deeply anaesthetized with 0.2% MS222 and perfused transcardially with saline, followed by 4% paraformaldehyde in PBS (pH 7.3). The brains were then removed and post-fixed in the same fixative overnight. Fixed brains were washed with PBS and immersed in 0.1 M PBS containing 20% sucrose overnight. Serial frontal sections (20-μm thickness) or mirror-image neighbouring frozen sections were cut on a cryostat (CM-3050-S, Leica Microsystems, Wetzlar, Germany), mounted onto MAS-coated glass slides (Matsunami Glass, Osaka, Japan), and stored at 20 °C.

The sections were stained either with one of the antibodies against GnRH3 (×7000) and IGF-1R (MBS8235569, ×1000, rabbit polyclonal antibody, MyBioSource. Com., San Diego, CA, USA), the latter of which recognizes human, rat, bovine, chicken/birds, and zebrafish antigen according to the manufacture instruction. Since these two antibodies were polyclonal antibodies raised in rabbits, a pair of mirror-image frozen sections was immunostained separately with those antibodies. The sections were incubated overnight with one of the antibodies. After washing with PBS, they were incubated with either Alexa Fluor 488- or 555-conjugated goat anti-rabbit IgG (500×, ThermoFisher Scientific, Ltd.). Hoechst 3334 solution (1000×) was used for nuclear staining. Finally, coverslips were mounted with ProLong Gold antifade reagent. Stained slices were observed using a confocal laser microscope.

### Real-time qRT-PCR analysis

Nine animals were used in this experiment. Brain slices at the TN were cut into left and right halves, which were used for the 11-KT (10 nM) and control groups, respectively. Thus, 11-KT-treated and control slices originated from the same fish. The RNA samples were collected from the slices 1 h after the treatment. Reverse transcription and real-time qRT-PCR were performed as reported previously^53,54^ (Tsutiya et al.^54,55^). Cytoskeletal *β-actin* mRNA was used as a reference for qRT-PCR assays. The primer pairs used are as follows; CTGTGGAGAGCGAGGCTTTT and TTGGGAGTCTTGACAGGTGC for *Igf-1*, and AGCCAACAGGGAGAAGATG CCGGAGTCCATGACGATAC for *β-actin*.

### Statistical analysis

Numerical data are presented as means ± standard error of the mean. The significance of differences between the control and experimental groups (Fig. 2, 3, and 4) was determined by the Student’s paired *t*-test, where *p* < 0.05 was considered statistically significant. In these experiments, the half brain slices for experimental and control groups originated from the same animals. For the control groups, the solvent was added to the medium at the same concentration as the corresponding experimental group.

In the experiments involving treatments with 11-KT and/or inhibitors (Fig. 5, 7, and 8), the effect of two factors (11-KT and the inhibitor treatments) on the number of GnRH3 neurons was analyzed by a two-way analysis of variance (ANOVA). When an interaction between the two factors was significant, the Tukey-Kramer test was used as a post-hoc test. In both cases, *p* < 0.05 was considered statistically significant. Because two-way ANOVA and commonly used post-hoc tests such as the Tukey test are not suitable for the comparison of individuals with the correspondence, we used a paired *t*-test for the statistical analysis of the numbers of GnRH3 neurons in the half brain slices originating from the same animal. Furthermore, we used Bonferroni correction to adjust the alpha (α) level for a family of statistical tests so that we control the probability of committing a type I error. The formula for a Bonferroni Correction is as follows: α new = α original / n. Paired *t*-test was conducted two times ('control' group *vs* 'inhibitor' group; '11-KT' group *vs* '11-KT and inhibitor' group) for each inhibitor experiment. Thus, *p* < 0.025, which was calculated from 0.05/2, was considered statistically significant in Figs. 5, 7, and 8.

## Data Availability

The data that support the findings of this study are available from the corresponding author upon reasonable request.
